# Clinical Experiences with Closed Incisional Negative Pressure Wound Treatment on Various Anatomic Locations

**DOI:** 10.7759/cureus.8849

**Published:** 2020-06-26

**Authors:** Burak Ozkan, Nilgun Markal Ertas, Ulas Bali, Cagri A Uysal

**Affiliations:** 1 Plastic and Reconstructive Surgery, Baskent University Faculty of Medicine, Ankara, TUR; 2 Plastic Surgery, Celal Bayar Üniversitesi, Manisa, TUR

**Keywords:** closed incisional negative pressure wound therapy, wound management, vac, wound dehiscence

## Abstract

Background

Closed incisional negative pressure wound treatment (ciNPWT) is one of the promising methods for the prevention of complications in surgical incisions. The mechanisms of ciNPWT have previously been elucidated and in this series, we demonstrate various, as of yet, underreported uses for the technology. Our aim is to share our experience with ciNPWT on various anatomic sites with novel indications.

Materials and methods

ciNPWT was used in 24 patients. The mean age was 49.6. All the incisions were sutured, clean, and non-infected. Patients’ sex, age, comorbidities, anatomic location of the wound, and the indications for ciNPWT were recorded.

Results

The mean number of applications was three per patient. One suture dehiscence after one session of ciNPWT was encountered in a flap donor site of an infant operated for meningomyelocele. Late-term seroma and hematoma formation were encountered in two patients. No surgical site infection, wound dehiscence, and ciNPWT related complications were seen in other patients. The majority of the applications were on the trunk, lower extremity, pelvis, upper extremity, and scalp respectively. Indications for ciNPWT utilization were preventing dehiscence, seroma, and hematoma formation in the majority of the patients.

Conclusion

ciNPWT is reliable and effective in the prevention of post-operative wound dehiscence and surgical site infections. It can be used safely in various locations and different indications for preventingcomplications such as preventing dehiscence in revision surgeries, cerebrospinal fluid (CSF) fistula formation in the scalp, and wound breakdown in chronic corticosteroid use,

## Introduction

Since its description in the early 90s, negative pressure wound therapy (NPWT) has revolutionized the conventional approach to the treatment of large and chronic wounds [1]. NPWT promotes wound healing with its proven effects such as an increase in blood flow, promoting the formation of granulation tissue and a decrease in edema, bacterial burden, and metalloproteinases [2-5]. With the advances of technology and research on vacuum effect on wounds, various types of sponges or devices for NPWT has been developed with different modalities such as installation and closed incisional vacuum system [6]. 

Closed incisional negative pressure wound treatment (ciNPWT) is a technology-based dressing designed to cover and support clean, closed incisions. This system maintains an isolated and sterile environment in closed incisions with NPWT principles. Studies showed that ciNPWT decreases the edema and pressure on the applied tissue and increases blood flow [7,8]. Thus lower rates of wound dehiscence were reported especially with high-risk patients [9]. ciNPWT provides lower surgical site infections, wound dehiscence, and lower costs with a shorter hospital stay when used in appropriately selected patients.

Promising outcomes have been reported in abdominoperineal wounds, sternal wounds, post arthroplasty and joint replacement procedures, post-bariatric dermo lipectomy cases, extremity fractures, and fasciotomy closure [10-12]. 

In this study, we share our experience with ciNPWT on various anatomic sites as well as complications in patients with novel indications.

## Materials and methods

Between October 2019 and February 2020, ciNPWT was used in a total of 24 patients. The mean age was 49.6. Body mass indexes (BMI) and comorbidities were recorded except in pediatric patients. All incisions were sutured, clean, and non-infected. Patients who had excessive tension on incisions such as flap donor sites, fasciotomy closures, and who had a medical history of obesity (BMI>30), chronic corticosteroid use, coagulation disorders were considered as high risk for complication and ciNPWT were utilized in incisions of high-risk patients in primary cases. Additionally, ciNPWT was used in patients who had previous surgeries and wound-related complications in the abdominopelvic region, trunk, extremities, and head respectively. 

Application of closed incisional vacuum treatment

After layered closure of the operation site (flap donor site, defect closure site), the incision was cleaned and dried. A special sponge with suction tube and silicone adhesive (Uno, Genadyne, USA) was measured along the length and widthwise of the suture line and applied to the incision in the area of greatest tension. The sponge was secured with multiple transparent adhesive tapes. Colostomy paste (Adapt Paste; Hollister International, Libertyville, IL) was used in special areas such as web spaces and intergluteal fold, adjacent to ostomies in order to avoid pressure fall or air leakage. Borders of the adhesive tapes were covered with colostomy paste. Suction pressure was set to continuous -30 mmHg for infants and -70 mmHg for adults. The dressing was changed once every three days.

## Results

The mean number of sessions for ciNPWT was three (1-5). Mean BMI was 28.1 (21-33). The location of the wounds was on the trunk in 13 patients, on the lower extremity in five patients on the pelvis in three patients, on the upper extremity in two patients and head in one patient. Early wound dehiscence was encountered in a flap donor site of a newborn patient who had been operated for meningomyelocele. The open wound was treated with conventional vacuum-assisted closure (VAC) for promoting secondary healing which eventually healed without complication after two applications. Hematoma formation was encountered in a patient operated for revision hip arthroplasty who had a medical history of liver transplantation and coagulation disorder. Late seroma was seen in an inguinal dissection site. Two patients who had ciNPWT on the scalp and sternum complained of pain and discomfort. We experienced maceration along the incision line in two patients after the first session that did not lead to dehiscence. The list of the patients is provided in Table 1.

**Table 1:** List of patient characteristics and closed incisional negative pressure therapy indications and outcomes. CSF: cerebrospinal fluid; MCA: middle cerebral artery; COPD: chronic obstructive pulmonary disease

Patient	Age	Sex	Location	Diagnose	Indication	Number of Sessions	Complication	BMI	Comorbidity
1	63	Female	Upper Extremity	Tertiary Brachioplasty	Prevention of edema and hematoma formation and suture dehiscence	4	-	33	Diabetes Lupus Erythematosus Pulmonary Thromboembolism Chronic steroid usage
2	25	Female	Head	Intracranial hemorrhage	Prevention of CSF leakage	3	-	25	Cystinosis Chronic Renal Failure Renal Transplantation MCA aneurysm Intracranial hemorrhage Hypothyroidism
3	69	Female	Trunk	Endometrium cancer	Isolation from stoma and dehiscence prevention	2	-	33	Cardiac arrhythmia
4	66	Male	Lower extremity	Diabetic foot	Fasciotomy closure	2	-	26	Diabetes Mellitus Peripheric arterial disease
5	27	Male	Pelvis	Gluteal cyst excision	Isolation from fecal contamination and maceration	2	-	21	-
6	3	Male	Trunk	Hemangioma excision	Prevention of hematoma formation after drain removal	1	-	-	-
7	13 days	Female	Trunk	Meningomyelocele repair	Prevention of flap donor site dehiscence	1	Dehiscence	-	-
8	10	Male	Lower Extremity	Benign tumor excision	Prevention of plantar dehiscence Early mobilization	2	Maceration	-	
9	61	Male	Trunk	Sternal osteomyelitis	Prevention of dehiscence and surgical site infection	2	-	30	Cardiac arrest Hypertension COPD
10	42	Male	Trunk	Anterolateral thigh free flap donor site	Prevention of dehiscence	2	-	32	-
11	47	Female	Pelvis	Pelvic exenteration	Prevention of dehiscence and surgical site infection	3	-	30	Clear cell over ca
12	52	Female	Trunk	Deep inferior epigastric artery perforator flap donor site	Prevention of dehiscence	3	-	26	Breast cancer
13	42	Female	Trunk	Abdominoplasty	Prevention of dehiscence	2	-	32	Diabetes Mellitus
14	63	Female	Trunk	Hysterectomy	Draining fat necrosis and prevention of suture dehiscence	5	-	33	Diabetes Mellitus Hypertension
15	58	Female	Pelvis	Pelvic Exenteration	Prevention of dehiscence and seroma formation	4	-	28	-
16	72	Female	Lower extremity	Revision hip arthroplasty	Prevention of dehiscence, hematoma	5	Hematoma formation	30	Liver transplantation
17	73	Male	Trunk	Inguinal lymph dissection	Prevention of dehiscence and seroma	3	Late seroma formation	35	Melanoma Morbid obesity
18	58	Male	Lower extremity	Diabetic foot	Prevention of dehiscence at Transmetatarsal amputation incision	3	-	23	Diabetes Peripheric arterial disease Chronic Kidney Failure
19	78	Male	Lower extremity	Diabetic foot	Prevention of dehiscence at Transmetatarsal amputation incision	2	-	25	Diabetes Peripheric arterial disease Chronic Kidney Failure
20	82	Male	Trunk	Spinal instrumentation revision	Prevention of dehiscence	5	-	22	Multiple myeloma
21	61	Male	Trunk	Spinal instrumentation revision	Prevention of dehiscence	4	-	29	Diabetes
22	33	Female	Trunk	Nipple areolar complex necrosis	Epithelization	5	-	23	-
23	63	Male	Upper extremity	Radial forearm donor site revision	Prevention of dehiscence	2	-	24	Diabetes
24	44	Female	Trunk	Spinal instrumentation revision	Prevention of dehiscence	3	-	32	Diabetes Morbid obesity

Patient 1

**Figure 1:**
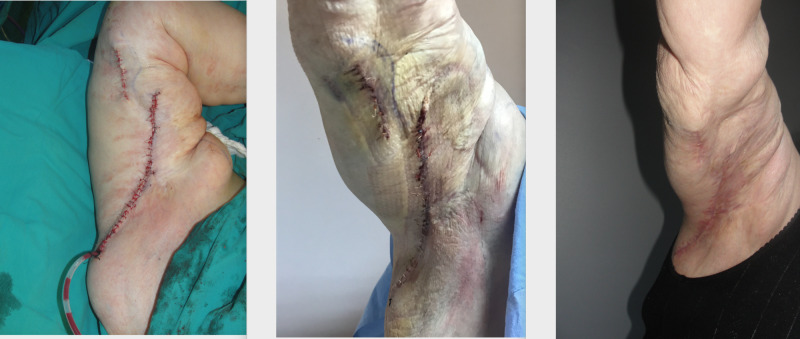
Intra-operative, early and late post-operative images of the patient operated for brachial dermo lipectomy; four sessions of ciNPWT were used right after the operation. ciNPWT: closed incisional negative pressure wound treatment

A 63-year-old female patient presented to our outpatient clinic with disfiguration of bilateral arms. She had a history of two brachioplasties which had been complicated and required healing by secondary intentions. The patient had diabetes, lupus erythematosus, and pulmonary thromboembolic history. Due to these comorbidities, anticoagulant treatment could not be stopped. She had dermal atrophy and fragile skin quality as a consequence of chronic steroid usage. After scar revision dermo lipectomy, ciNPWT was applied immediately in an effort to prevent hematoma formation, skin breakdown, and early wound dehiscence in addition to suction drains after the removal of drains. Daily output was over 50 ml for three days in the vacuum canister and drains were below 20 ml. Drains were removed on the fourth day and ciNPWT was applied in four sessions (12 days) and no complication was encountered. Intra-operative, early and late post-operative images of the patient are demonstrated in Figure 1.

Patient 2

**Figure 2:**
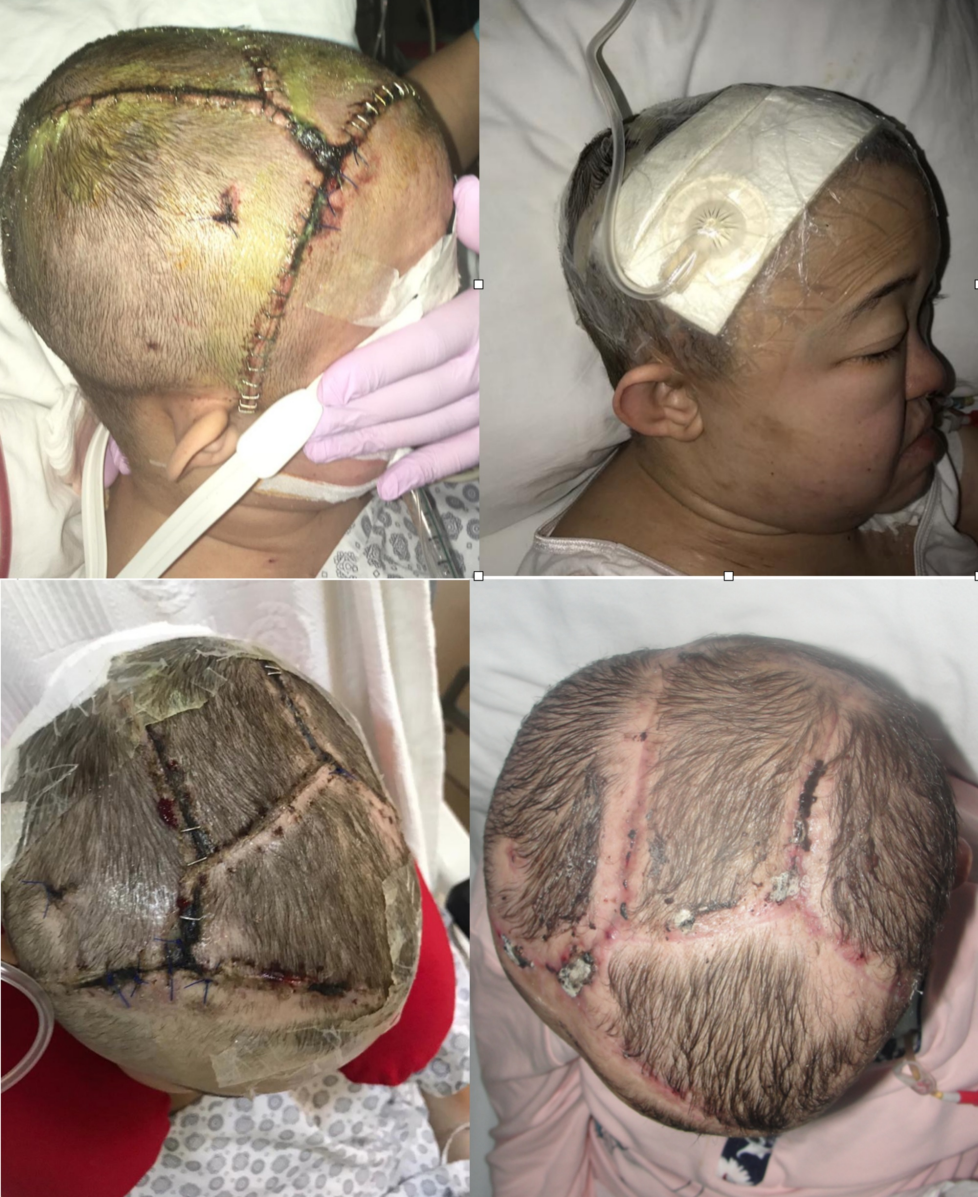
(Top left) Early post-operative image and (top right) application of ciNPWT to the scalp. The patient had decompression surgery four times; formation of CSF fistula was prevented in spite of CSF accumulation under the scalp after drain removal (bottom left). Post-operative image of the patient, in the second month, after four sessions of ciNPWT (bottom right). ciNPWT: closed incisional negative pressure wound treatment; CSF: cerebrospinal fluid

A 25-year-old female was consulted at the neurosurgery department. She had spontaneous rupture of intracranial aneurysms and undergone decompression surgery for intracranial hemorrhages four times. Her defect included dura mater and cranial bone. Neurosurgeons removed suction drains in post-operative seventh day. After drain removal, cerebrospinal fluid (CSF) collection developed under scalp flaps. Because of the high risk for CSF leakage or fistula, a decision was made to place an incisional VAC which was not a standard indication for ciNPWT utilization. The pressure was adjusted as staying over intracranial pressure limits while preventing uncontrolled CSF suction and was set as continuous -30 mmHg. ciNPWT was closely monitored in case of CSF aspiration. She complained of pain that responded to analgesics while she received continuous negative pressure. Therapy was used in four sessions (12 days) until suture removal. No dehiscence, CSF leakage or wound-related complications were encountered. Early and late post-operative images are demonstrated in Figure 2.

## Discussion

The benefits of ciNPWT are based on VAC effects on wound healing. It has been shown that the blood flow in the wound edges is increased while tissue edema and the tension of the suture line is decreased [13]. Unlike traditional gauze dressings, the surgical site is isolated and vacuumed under sub-atmospheric pressures in ciNPWT to prevent surgical site infections and wound breakdown. Lower rates of dehiscence were reported in multiple studies with ciNPWT utilization, especially in high-risk locations and patients [14]. 

The abdominopelvic region is a high-risk location for post-operative wound breakdown [15]. Perfusion of the wound edges is diminished due to prolonged traction in gynecologic operations such as pelvic exenterations. Patients with obesity are also at high risk for dehiscence. It has been shown that abdominal fat has a lower perfusion rate that makes them prone to fat necrosis after abdominal surgeries [16]. The existence of colostomy or ileostomy nearby the surgical site is an additional risk for surgical site contamination and infection. ciNPWT can maintain a sterile isolated environment and reduces the risk of contamination [17]. The majority of our cases had surgeries (5/15) in the abdominopelvic region. Although these patients had high BMIs and prolonged operative time, we did not encounter a surgical site infection or wound breakdown. Another observation that we have is the role of ciNPWT in draining superficial necrotic fat debris after drain removal. Surgical drains are often placed in deeper cavities or suprafasial planes to remove blood, seroma but they are not sufficient in the extraction of fat necrosis in superficial levels in obese patients. The fat necrosis causes discharge through the suture line and prolonged exposure of fat debris may lead to maceration and wound dehiscence in conventional wound dressings. ciNPWT can remove this exudate and discharge while maintaining the continuity of the incisions. Five to seven days or two sessions are suggested for optimum gain from ciNPWT [18]. 

**Figure 3:**
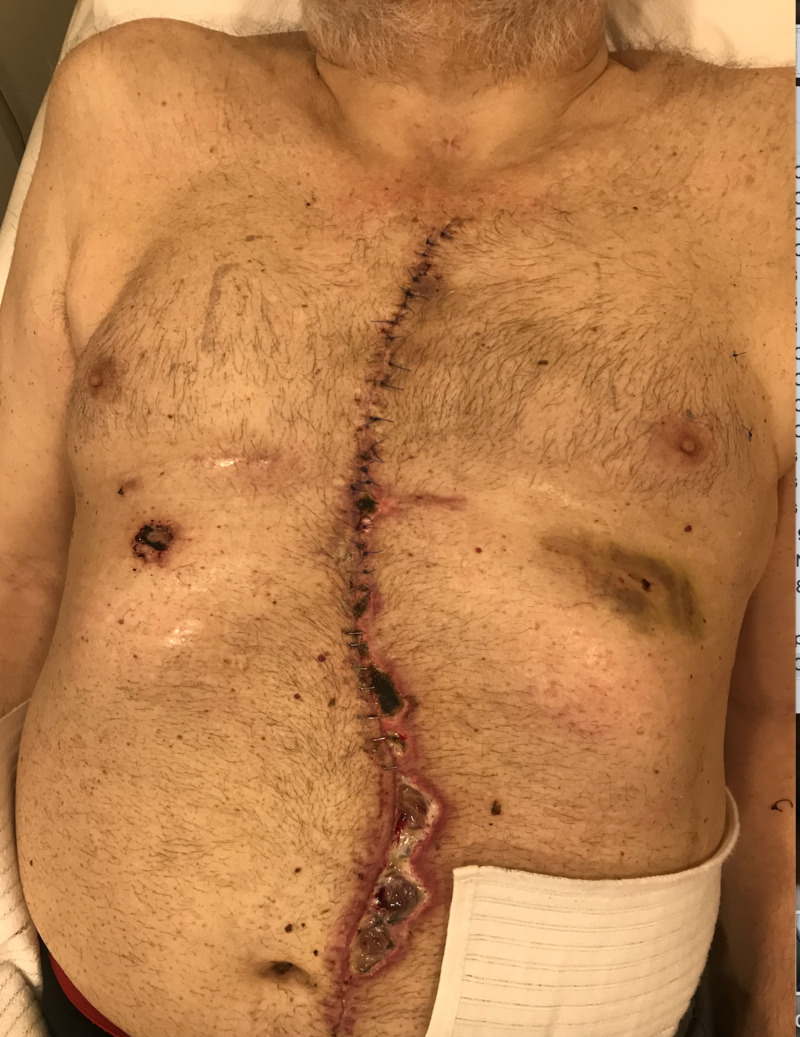
Post-operative first month image of a sternal osteomyelitis case reconstructed with rectus abdominis muscle flap. Two sessions of ciNPWT was used for sternal incisions. While no problem was observed in the sternum, wound breakdown was observed in the abdominal region. ciNPWT: closed incisional negative pressure wound treatment

Sternal incisions due to cardiovascular surgery or sternal osteomyelitis management are other indications for ciNPWT utilization. The blood circulation of the sternum is often diminished after bypass surgery due to losing the internal mammary artery. This can cause a breakdown in sternal wounds or persistent osteomyelitis to fatal mediastinitis [19]. Sequestration of sternal clean incisions with ciNPWT is recommended in many studies. Jennings et al. found a statistically significant lower surgical site infection in the usage of ciNPWT [20]. We had a sternal osteomyelitis case after excessive resection and reconstruction with pedicled rectus abdominal muscle flap. We used two sessions of (six days) ciNPWT to the sternal incision and we did not utilize ciNPWT to the abdomen that has lower tension on the skin edges. Interestingly, we confronted abdominal dehiscence in the post-operative first week while no complication was seen in the sternal area (Figure 3).

Fragile skin texture as a result of chronic steroid usage and connective tissues diseases is considered as a relative contraindication to VAC applications [21]. However, we used ciNPWT after brachial dermolipectomy in a patient with lupus and chronic steroid use history without complication. Our observation was lesser edema and ecchymoses in the incisions lines and early removal of drains. Similar benefits of incisional vacuum therapy were reported in post-bariatric dermo lipectomy cases in the literature [22]. 

Novel indications in our cases were preventing cerebrospinal skin fistula on the scalp and application of ciNPWT to flap donor site in a newborn with myelomeningocele. There are reports about the employment of VAC on the cortical brain, in cases of the dura and calvarial defects in the literature [23,24]. But, any reports regarding the prevention of CSF fistula were published. We have shown that accumulation of CSF under the scalp in terms of dural and calvarial defects can be managed without fistula formation by closed incisional vacuum treatment. We believe that this case may be a clinical example of the proven effect of mechanical strength that ciNPWT provides to wound edges [25]. Our recommendation is keeping the negative pressure below 40 mmHg for counteracting headache and uncontrolled CSF aspiration to the canister. Reports on the application of VAC in infants are rare [26]. After the closure of the meningomyelocele defect with perforator flap, we closed the donor site of the flap primarily. After one session of incisional vacuum treatment on the flap donor site with 30mm Hg, treatment was stopped. Wound dehiscence was encountered on the post-operative fifth day on the flap donor site. We think that one session of ciNPWT may not be effective to prevent complications on infants likewise adults. Nonetheless, more cases and experience is needed to conclude the effect of closed incisional wound treatment on infants and scalp CSF fistula prevention. 

As a summary, our experience with ciNPWT was encouraging for decreasing tension on the suture lines, maintaining lesser edema, hematoma and seroma, earlier drain removal, prevention of contamination from colostomy/ileostomy in abdominal sutures, draining fat necrosis debris, lesser dressing change, prevention of CSF fistula formation, wound dehiscence and surgical site infections. Heterogeneity of the cases and the absence of comparison with non ciNPWT treatment are the weak parts of our study. Although we believe the benefits can be better understood with cost analyses studies with large cohorts, our experiences with ciNPWT can contribute to physicians in the decision making of ciNPWT usage.

## Conclusions

The anatomic location of the surgery, the tension on the wound edges, and comorbidities of the patient such as obesity, diabetes, the vascular status of the wound site are the major predictors of the possible wound breakdown. Thus, an individual approach is needed to encounter lower rate of complications. The surgeon has to determine the risks and take precautions for the post-operative period. According to literature reviews and our experience, we believe ciNPWT can be safely used in various locations and in different indications for preventing complications.

## References

[1] Fleischmann W, Strecker W, Bombelli M, Kinzl L (1993). Vacuum sealing as a treatment of soft tissue damage in open fractures [Article in German]. Unfallchirurg.

[2] Argenta LC, Morykwas MJ (1997). Vacuum-assisted closure: a new method for wound control and treatment: clinical experience. Ann Plast Surg.

[3] Morykwas MJ, Argenta LC, Shelton-Brown EI, McGuirt W (1997). Vacuum-assisted closure: and basic foundation. Ann Plast Surg.

[4] Banwell PE (1999). Topical negative pressure therapy in wound care. J Wound Care.

[5] Chen SZ, Li J, Li XY, Xu LS (2005). The experimental study of the effects of vacuum-assisted closure on edema and vessel permeability. Asian J Surg.

[6] Conde-Green A, Chung TL, Holton LH III (2013). Incisional negative-pressurewound therapy versus conventional dressings following abdominal wall reconstruction: a comparative study. Ann Plast Surg.

[7] Wilkes RP, Kilpad DV, Zhao Y, Kazala R, McNulty A (2012). Closed incision management with negative pressure wound therapy (CIM): biomechanics. Surg Innov.

[8] Atkins BZ, Tetterton JK, Petersen RP, Hurley K, Wolfe WG. (2011). Laser Doppler flowmetry assessmentof peristernal perfusion aftercardiac surgery: beneficialeffect of negative pressure therapy. Int Wound J.

[9] Blackham AU, Farrah JP, McCoy TP, Schmidt BS, Shen P, Allen G (2013). Prevention of surgical site infections in high-risk patients with laparotomy incisions using negative-pressure therapy. Am J Surg.

[10] Pachowsky M, Gusinde J, Klein A (2012). Negative pressure wound therapy to prevent seromas and treat surgical incisions after total hip arthroplasty. Int Orthop.

[11] Grauhan O, Navasardyan A, Hofmann M, Muller P, Stein J, Hetzer R (2013). Prevention of poststernotomy wound infections in obese patients by negative pressure wound therapy. J Thorac Cardiovasc Surg.

[12] Wiegering A, Dietz UA, Corteville C (2017). Impact of incisional negative pressure wound therapy on perineal wound healing after abdominoperineal rectum extirpation. Int J Colorectal Dis.

[13] Timmers MS, Le Cessie S, Banwell P, Jukema GN (2005). The effects of varying degrees of pressure delivered by negative-pressure wound therapy on skin perfusion. Ann Plast Surg.

[14] Stannard JP, Volgas DA, McGwin G III, Stewart R, Obremskey W, Moore T, Anglen J (2012). Incisional negative pressurewound therapy after high-risk lower extremity fractures. J Orthop Trauma.

[15] Pauli EM Krpata DM, Novitsky YW, Rosen MJ (2013). Negative pressure therapy forhigh-risk abdominal wall reconstruction incisions. Surg Infect.

[16] Dragu A, Schnürer S, Horbach T (2012). Evaluation of intra-operative abdominal wall perfusion in post-bariatric abdominal dermolipectomy. Obes Facts.

[17] Lewis LS, Convery PA, Bolac CS, Valea FA, Lowery WJ, Havrilesky LJ (2014). Cost of care using prophylactic negative pressure wound vacuum on closed laparotomy incisions. Gynecol Oncol.

[18] Matatov T, Reddy KN, Doucet LD, Zhao CX, Zhang WW (2013). Experience with a new negative pressure incision management system in prevention of groin wound infection in vascular surgery patients. J Vasc Surg.

[19] Wu RT, Sumpio BJ, Miller S, Sumpio BE (2019). Use of closed-incision negative-pressure therapy: cardiothoracic and vascular surgery. Plast Reconstr Surg.

[20] Jennings S, Vahaviolos J, Chan J, Worthington MG, Stuklis RG (2016). Prevention of sternal wound infections by use of a surgical incision management system: first reported Australian case series. Heart Lung Circ.

[21] Venturi ML, Attinger CE, Mesbahi AN, Hess CL, Graw KS (2005). Mechanisms and clinical applications of the vacuum-assisted closure (VAC) device. Am J Clin Dermatol.

[22] Dragu A, Schnürer S, Unglaub F, Wolf MB, Beier JP, Kneser U, Horch RE (2011). Wide topical negative pressure wound dressing treatment for patients undergoing abdominal dermolipectomy following massive weight loss. Obes Surg.

[23] Prince N, Blackburn S, Murad G (2015). Vacuum-assisted closure therapy to the brain: a safe method for wound temporization in composite scalp and calvarial defects. Ann Plast Surg.

[24] Pereira S, Malta W, Canha A, Polonia J (2017). Vacuum-assisted closure therapy after resection of giant basal cell carcinoma of the scalp. J Surg Case Rep.

[25] Kilpadi DV, Lessing C, Derrick K (2014). Healed porcine incisions previously treated with a surgical incision management system: mechanical, histomorphometric, and gene expression properties. Aesthetic Plast Surg.

[26] Banda CH, Narushima M, Ishura R, Fujita M, Furuya M (2018). Local flaps with negative pressure wound therapy with secondary reconstruction of myelomeningocele wound necrosis. Plast Reconstr Surg Glob Open.

